# Cancer control in Africa: surgery

**DOI:** 10.3332/ecancer.2019.943

**Published:** 2019-07-25

**Authors:** David J Galloway

**Affiliations:** Royal College of Physicians and Surgeons of Glasgow, 232-242 St Vincent Street, Glasgow G2 5RJ, UK

**Keywords:** surgery, surgical procedure, surgical plan, oncology, Africa, specialist care

## Abstract

Surgery is the mainstay of cancer treatment and lack of surgical treatment is a major driver in holding back optimal cancer care. Surgery is essential for global cancer care in all resource settings. Of the estimated 18.1 million new cases of cancer in 2018, over 80% of cases will need surgery, some several times. Many patients throughout the world do not have access to cancer surgery. Many of the key adjunct treatment modalities for cancer surgery—e.g., anaesthesia, pathology and imaging—are also inadequate. Solutions are necessary and should include better-regulated public systems, international partnerships, super-centralisation of surgical services, novel surgical clinical trials and new approaches to improve quality and scale up cancer surgical systems through education and training. Delivery of safe, affordable and timely cancer surgery to all must be at the heart of global and national cancer-control planning.

There has rarely been a more clearly articulated requirement for a global commitment to respond to the desperate need for enhanced surgical, anaesthetic and obstetric care in low-income countries such as most of the African nations.

In the context of delivering cancer treatment services, this is even more emphatic. Surgery is not only the primary treatment option, being the only curative treatment option for virtually all common solid cancers, but surgical skill is also an important component of the diagnostic process. However, surgical care cannot function in a vacuum. Infrastructure development is an essential component of developing services.

Surgical care has been described as the ‘neglected stepchild of global health’ [1]. Clinical conditions requiring surgical and anaesthetic care amount to 30% of the global disease burden and some 70% of the world’s population do not have access to safe, affordable, surgical and anaesthetic care when it is required [2].

The importance of surgery as an international health priority was clearly recognised in the World Health Assembly by its adoption of Resolution 68.15 [3].

Not only is the population of the African continent increasing more rapidly than any other, life expectancy is also increasing so it is inevitable that the current considerable cancer burden will only become more demanding. Great emphasis has been placed on the burden and challenge of communicable diseases in Africa. It is true that some of these have a direct role in contributing to the burden of malignant disease. However, the major killing conditions are usually considered to be malaria, HIV/AIDS and TB. In reality, however, around four times as many individuals die of conditions which can be addressed surgically and cancer cases comprise many of these. Detailed information concerning surgical oncology provision is rudimentary. While specialist surgeons are now developing a specific practice in cancer treatment, it is not helpful to try to draw an analogy with the way specialist surgical care has developed in Western nations. It is, however, possible to draw some inferences from data pertaining to broader surgical service delivery in Africa.

An attempt to currently and accurately define the number of surgical oncologists in African countries is almost impossible. There are, however, data available to allow comparison of national surgical activity although it is not detailed enough to provide any useful detail for cancer surgery. For example, the evidence suggests that the total rate of surgical procedures per head of population ranges from 43 per 100,000 for Ethiopia compared with 15,280 for the United Kingdom. For the UK, 6,552 (42.8%) of these were classified as cancer-specific. For other selected African nations, the surgical rates per 100,000 were: 3,321 for Mali, 850 for Rwanda, 722 for Zambia, 331 for Liberia, 241 for Uganda and 53 for Chad [4]. In a tri-national study [5], featuring information from Tanzania, Uganda and Mozambique, non-maternity operations comprised up to 60% of the surgical procedures performed at district level. The majority of these were classified as major operations. In Tanzania, almost 80% of the hospitals could provide basic care but only 51% were considered capable of offering a safe service, the main drawbacks being the lack of appropriate personnel and equipment. The logic of maintaining specialist care in central teaching hospitals is undeniable. In the district and rural context, it is more practical to focus on less-complex surgery and while this may prove to be attractive for practical reasons of both cost and sustainability, it also provides a solid basis for addressing the clinical demand. Furthermore, recognition of the value of surgical care (even that provided by personnel, such as clinical officers, who may not be formally trained as surgeons but can nevertheless support a service focussed on early detection and diagnostic procedures).

While it is a laudable goal to scale up provision by providing a centralised specialist service in the major hospitals, the reality of life in rural Africa is that the prospect of requiring travel and accommodation to receive such specialist care may be well beyond the means of patients whose local presence is vital to provision for their families.

Clinical experience in Africa confirms that late presentation and advanced pathology are the rule and the resulting challenge in made greater by the inadequacy of oncological support with respect to the availability of chemotherapy drugs (of the 44 chemo-therapeutic agents listed by the WHO for cancer treatment, the median number available and adapted for inclusion in national formularies in the African region is 15) [6]. Similarly, there is a desperate shortage of radiation therapy—some 3 million patients per treatment unit is the current ratio for the 1.2 billion population of Africa compared to 400–500 per treatment unit being the desired provision [7].

Any attempt to address the requirements for cancer care in Africa must urgently deal with these concerns. Care is necessarily complex and whilst elements of the process could be delegated to suitably trained non-medical personnel, the need for integrated teams of clinicians providing a multidisciplinary approach must remain the goal if any significant impact on outcome is to be expected.

So what are the practical options for addressing this scenario?

- There is clearly a need to increase the number of medical and surgical staff with the necessary training to contribute to service provision.
- It is highly desirable that indigenous African clinicians provide the mainstay of the developing workforce. Whilst opportunities to train clinicians may well involve international support, it is critical that the workforce be retained within Africa in the long term for the desired outcome of improved care to be realised. While that workforce is being developed, there will continue to be a need for external clinical support from international medical staff to provide service and training.
- There is a need to build infrastructure systems and recruit networks of specialists in medical and radiation oncology as well as palliative care, to provide the diverse levels of care necessary to make an impact on the devastating burden of cancer morbidity and mortality.
- There are opportunities to develop teams of non-medical staff to assist with elements of the diagnostic and indeed some therapeutic aspects of care. Clinical officers could, as an example, provide surgical diagnostic support by providing biopsy services under supervision. This could ease the burden of care delivery from overstretched surgical staff and improve the efficiency of case selection, potentially deselecting those patients who may not require a more complex pattern of care.
- Surgery must assume a more prominent role in treatment provision. To service this, there is a need to develop the provision of suitable infrastructure support and the availability of allied services including diagnostic radiology, blood transfusion, pathology services, chemotherapy and radiation oncology. Other approaches in tackling the cancer burden include the need to integrate and develop community care support and inevitably the need for good-quality end-of-life care.

## Conclusion

A significant priority for each African nation must now be to give careful consideration to the production of a realistic national surgical plan. The Lancet Commission [2] provided a template and some states have already led the way. In particular, it is encouraging to record the detail and resource implications included in the plan published by the Ministry of Health in Tanzania [8]. It could be taken as an exemplar. There is a need for African national governments to determine both realistic and imaginative solutions to tackle surgical need. A combined approach is needed to enhance a competent surgical workforce, recruit and retain other healthcare personnel together with the implementation of strategies to improve infrastructure and support services, especially in populous rural communities.

## Funding

This work is unfunded.

## Conflicts of interest

The author has no conflicts of interest to declare.
